# Prediction of Single-Event Effects in FDSOI Devices Based on Deep Learning

**DOI:** 10.3390/mi14030502

**Published:** 2023-02-21

**Authors:** Rong Zhao, Shulong Wang, Shougang Du, Jinbin Pan, Lan Ma, Shupeng Chen, Hongxia Liu, Yilei Chen

**Affiliations:** School of Microelectronics, Xidian University, Xi’an 710071, China

**Keywords:** deep neural network (DNN), FDSOI devices, single-event effect (SEE), drain transient current pulse

## Abstract

Single-event effects (SEE) are an important index of radiation resistance for fully depleted silicon on insulator (FDSOI) devices. The research into traditional FDSOI devices is based on simulation software, which is time consuming, requires a large amount of calculation, and has complex operations. In this paper, a prediction method for the SEE of FDSOI devices based on deep learning is proposed. The characterization parameters of SEE can be obtained quickly and accurately by inputting different particle incident conditions. The goodness of fit of the network curve for predicting drain transient current pulses can reach 0.996, and the accuracy of predicting the peak value of drain transient current and total collected charge can reach 94.00% and 96.95%, respectively. Compared with TCAD Sentaurus software, the simulation speed is increased by 5.10 × 10^2^ and 1.38 × 10^3^ times, respectively. This method can significantly reduce the computational cost, improve the simulation speed, and provide a new feasible method for the study of the single-event effect in FDSOI devices.

## 1. Introduction

The anti-irradiation performance of integrated circuits is an important index in circuit design. With the progress of technology, the anti-irradiation effect of fully depleted Silicon on insulator technology (FDSOI) device circuits becomes more and more complex [1,2,3,4]. The research on the anti-irradiation performance of FDSOI devices has become a hot topic in the industry. The anti-irradiation performance includes single-event effects (SEE), total-dose effects, displacement effects, and so on. Among them, an SEE is caused by the interaction between high-energy particles and microelectronic devices or circuits, which will affect the work of devices and even damage the circuits [5,6,7].

The traditional device research method is based on simulation software such as Silvaco, TCAD Sentaurus, and other simulation software [8,9,10,11]. For example, Bi J. et al. studied FDSOI devices using Sentaurus software. and first used SDE (SDE is a simulation tool of TCAD Sentaurus software) for device modeling and grid division. After establishing the correct model, the device should be used for physical function modeling before further analysis and research [11]. This process not only requires researchers to be familiar with FDSOI devices and simulation software but also requires a lot of time to establish the correct device model and physical simulation, which brings great inconvenience to device research. In addition, when using TCAD to simulate the single-event effect of multiple devices, there is a convergence problem that has not yet been solved at present.

In recent years, deep learning has been widely applied in the field of electronic design automation because deep learning can form more abstract high-level attribute features by combining low-level features and discovering distributed features of data [12,13,14,15,16]. Moreover, deep learning has the characteristics of simple operation and fast simulation speed, making it a very powerful research method in quantum mechanics, optical materials, nanostructures, and other fields. For example, Jing Chen et al. obtained an average error of less than 4% when using a deep neural network to predict SOI lateral power device breakdown [17]. Christian C. et al. applied deep learning to accelerate all-dielectric metasurface design, and the mean square error was only 1.16 × 10^−3^ [18]. Kashyap Mehta et al. show that complete FinFET current–voltage and capacitance–voltage curves can be predicted using machine learning [19]. These results demonstrate the advantages of deep learning in device research. However, they predicted that FinFET’s current–voltage and capacitance–voltage curves only used 250 sets of data [19], which is a relatively small range and can only be applied to a small part of the device parameter range.

In this paper, a deep learning method for predicting the SEE of FDSOI devices is proposed. By inputting different particle incident device conditions, the corresponding characterization parameters of SEE can be obtained very quickly and then the influence of particle incident conditions on SEE can be studied. We design two network models, one for the prediction of drain transient current pulse and the other for the prediction of drain transient current peak and the total collected charge. In the process of network training and network testing, we use such parameters as accuracy, mean square error, and goodness of fit to quantify network performance [20,21]. In addition, we also use some traditional machine learning methods to compare with the deep neural network model we designed, to demonstrate the advantages of deep neural networks. The results show that the trained network has a very good effect. At the same time, our prediction method provides a new possibility for device research.

## 2. Experimental

### 2.1. Device Structure

**Figure 1:**
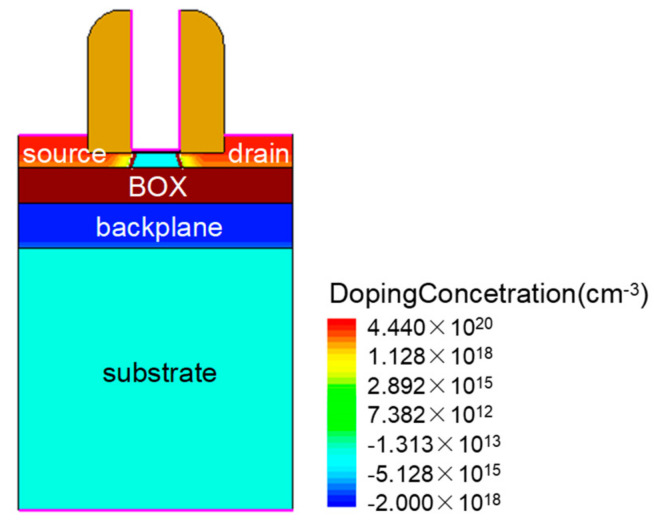
FDSOI device structure.

The FDSOI device structure we used is shown in Figure 1. The device uses silicon as the substrate and HfO_2_ as the gate oxide material. The source and drain of the device are doped with Gaussian, the doping concentration is N^+^ 4.4 × 10^20^ cm^−3^, and the body region and substrate are doped uniformly with P^−^ 1 × 10^15^ cm^−3^ and 1 × 10^14^ cm^−3^, respectively. To reduce the series resistance of the source and drain, the device model adopts the design of source and drain elevation [22,23]. In addition, a backplane layer is added under the device buried oxide (BOX) layer. The structural parameters of the device are shown in Table 1.

### 2.2. Dataset

**Figure 2:**
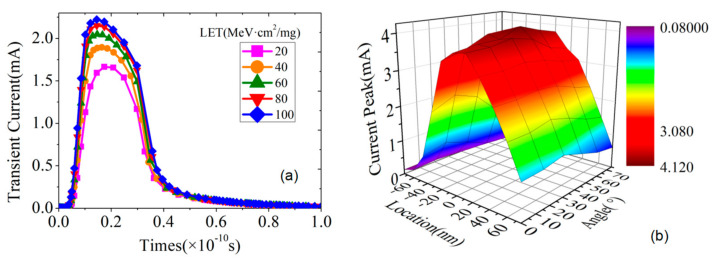
Influence of particle incident conditions on SEE: (**a**) is the effect of different LET values on the transient current pulse, and (**b**) is the effect of the position and angle of the particle incident on the device on the transient current peak.

**Figure 3:**
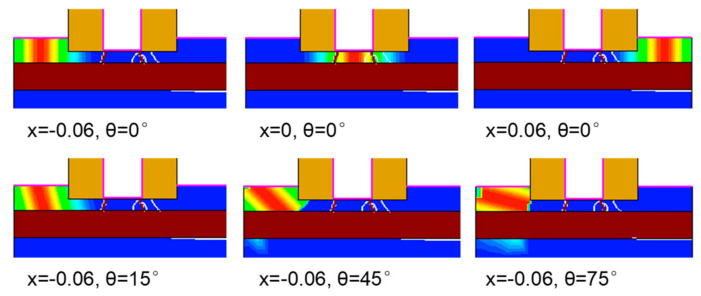
The charge density distribution of heavy ions at different positions and different angles of incidence.

To predict the SEE of FDSOI devices using deep learning, we must first determine the input features and output labels of the network. The input features should select the parameters that influence the SEE, and the output labels should be the characterization parameters of the SEE. Through TCAD simulation software, we selected the inputs including the linear transmission energy (LET) when the particle is incident on the device, particle incident position (x), the incident angle of the particle (θ), and the drain bias voltage (V_d_). The selected outputs include drain transient current pulse, drain transient current peak (I_0_), and total collected charge (Q_0_). Figure 2 shows the influence of the LET value on the transient current pulse and the influence of particle incident position and particle incident angle on the transient current peak. Figure 3 shows the charge density distribution of heavy ions at different positions and angles.

After determining the input and output parameters of the network, we obtain the data set by using TCAD simulation software. By changing the input parameters, 3300 data sets were obtained for training and testing the network model. The values of input data are shown in Table 2 (x = 0 is the center of the device, and the direction pointing to the drain is positive. θ is the angle between the particle incident direction and the vertical device surface).

To eliminate the adverse effects caused by singular sample data, we can use the MinMaxScaler function to normalize the data. That is, the data X is first centered at the minimum value and then scaled by the range (maximum–minimum) [24,25,26]. At this point, the data converges between [0, 1]. Normalized data are normally distributed. The normalized data are shown in Formula (1):

(1) $$x^{*}=\frac{x-\min\left(x\right)}{\max\left(x\right)-\min\left(x\right)}$$

The reason why input data should be normalized is that the essence of neural network learning is to learn the distribution of data. Once the distribution of training data and test data is different, the generalization ability of the network will be greatly reduced. On the other hand, once the distribution of each batch of training data is different, the network will have to learn to adapt to different distributions in each iteration, which will greatly reduce the training speed of the network [27,28,29].

We randomly divide the normalized input data according to the ratio of 6:2:2 to use as a training set, validation set, and test set for training the network.

### 2.3. Deep Neural Network Prediction of Drain Transient Current Pulse

**Figure 4:**
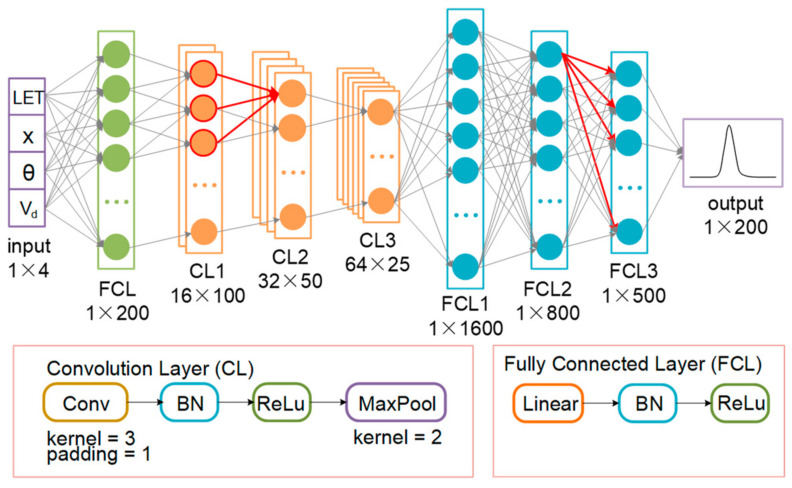
The network structure for predicting drain transient current pulses.

The network structure for predicting drain transient current pulses is shown in Figure 4. Four network input parameters are first passed through a full connection layer for input expansion, then through three convolutional layers, and then through three full connection layers. The input expansion layer extends the 1 × 4 input to 1 × 200. The full connectivity layer includes Linear, Batch Normalization (BN), and ReLu activation functions. The convolution layer consists of Conv, BN, ReLu, and Maxpool. The BN layer is to speed up the training speed of the network and alleviates the problem of gradient disappearance in the process of network training [30,31]. The ReLu activation function is designed to complete the nonlinear transformation of data, accelerate training speed, link gradient explosion, and gradient disappearance [32,33].

The output of this network is 200 points on the drain transient current pulse. To accurately describe the pulse curve, an average of 110 points were extracted in the time range of 0~0.03 ns, 60 points in the time range of 0.03~0.3 ns, and 30 points in the time range of 0.3~1 ns (the time range of the pulse is 0~1 ns).

### 2.4. Deep Neural Network Prediction of Drain Transient Current Peak and Total Collected Charge

**Figure 5:**
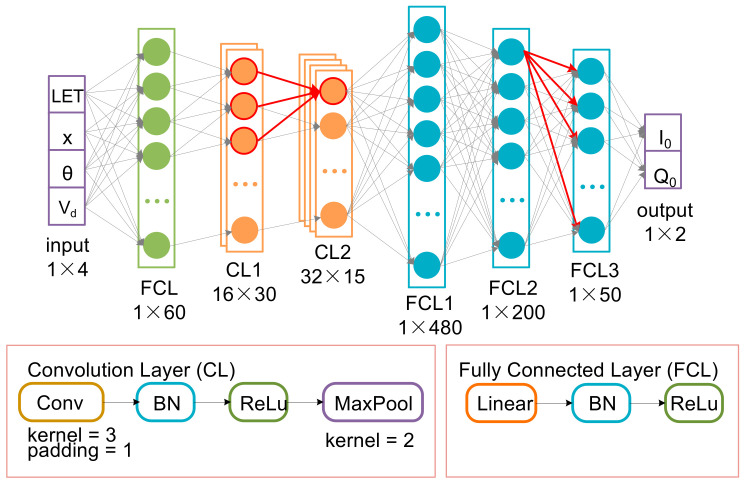
The network structure for predicting transient current peaks and total collected charges.

The network structure for predicting peak drain transient current and total collected charge is shown in Figure 5. The network structure is similar to that used to predict the drain transient current peak. The input is first passed through a fully connected layer for input expansion, then through two convolutional layers, and then through three fully connected layers. At this time, the input expansion expands the input of 1 × 4 to 1 × 60, and the data that finally enters the full connection is reduced correspondingly after one less layer of convolution, and the network output is two parameters. The rest of the structure remains largely unchanged. Some data used for network training and testing are shown in Table 3.

A common calculation method for I_0_ is shown in Equation (2):

(2) $$I_{0}=\frac{q^{2}\mu_{n}N_{a}N}{\varepsilon }x_{p}$$

where q is the electron charge, $\mu_{n}$ is the electron mobility, N_a_ is the doping concentration of the channel, N is the line density of the electron-hole pair, ε is the dielectric constant, and x_p_ is the width of the depletion region of the PN junction.

The representation of Q_0_ is shown in Equation (3), The time unit is ns:

(3) $$Q_{0}=\int_{0}^{1}\;I\left(t\right)\;dt$$

## 3. Results and Discussion

### 3.1. Results on Prediction of Ddrain Transient Current Pulse

**Figure 6:**
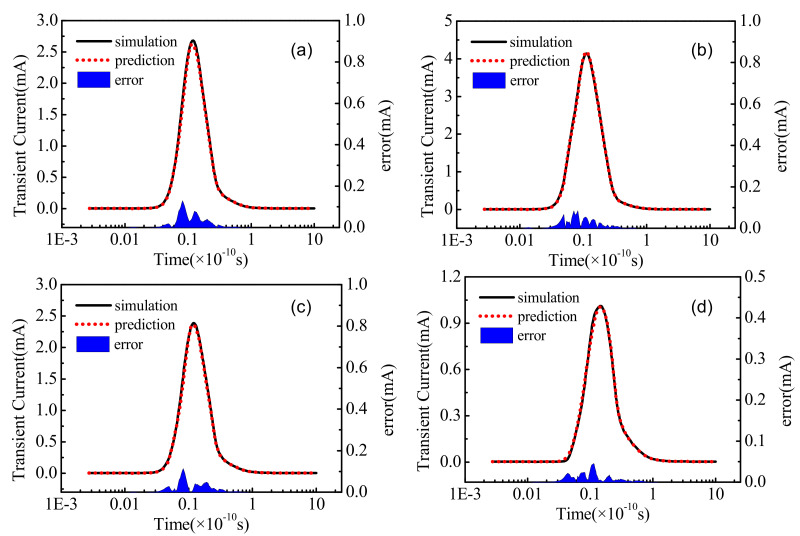
Comparison of simulation curve and prediction curve: (**a**–**d**) are the comparison results of the prediction curve and the simulation curve of four groups of transient current pulses randomly selected from the test set. The blue area in the figure is the difference between the two.

Figure 6 depicts the comparison of the simulation curve and prediction curve. Figure 6a–d are the comparison diagrams of six groups of simulation curves and predicted curves randomly selected from the test set. The red line is the curve predicted by the deep neural network, the blue line is the curve simulated by the TCAD software, and the blue area in the figure is the error value of the two curves. It can be seen from the figure that the fitting effect of the two curves is good and the error is small.

To further explain the predicted results of the curve, we adopt the parameter of the goodness of fit, which can reflect the degree of curve fitting. The maximum value of R^2^ is 1, and the closer R^2^ is to 1, the better the degree of curve fitting. Conversely, the smaller the value of R^2^, the worse the degree of the curve fit [34]. The calculation formula for R^2^ is shown in Equation (4), where y is the simulation value, $\overset{´}{y}$ is the predicted value, and $\bar{y}$ is the average value of the simulation value.

(4) $$R^{2}=\frac{\sum_{i=1}^{n}{\left({\overset{´}{y}}_{i}-\bar{y}\right)}^{2}}{\sum_{i=1}^{n}{\left(y_{i}-\bar{y}\right)}^{2}}$$

**Figure 7:**
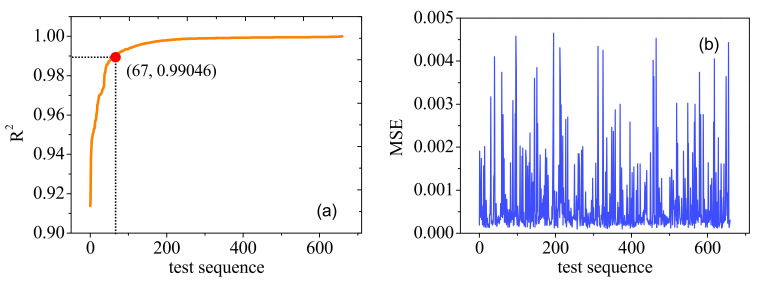
Results for the prediction of drain transient current pulse: (**a**) shows the goodness of fit of the test set, and (**b**) shows the MSE value of the test set.

We arranged the goodness of fit values of 660 test groups of curves in ascending order, and the result is shown in Figure 7a. It can be seen that R^2^ values are basically above 0.94, and curves with goodness of fit values above 0.99 account for 90%; the average goodness of fit of the curves is 0.9956. Figure 7b is the mean square error of each curve in the test set. It can be seen that the MSE values are all below 0.005, and the average MSE value for the 660 curves in the test set was 0.00068.

**Figure 8:**
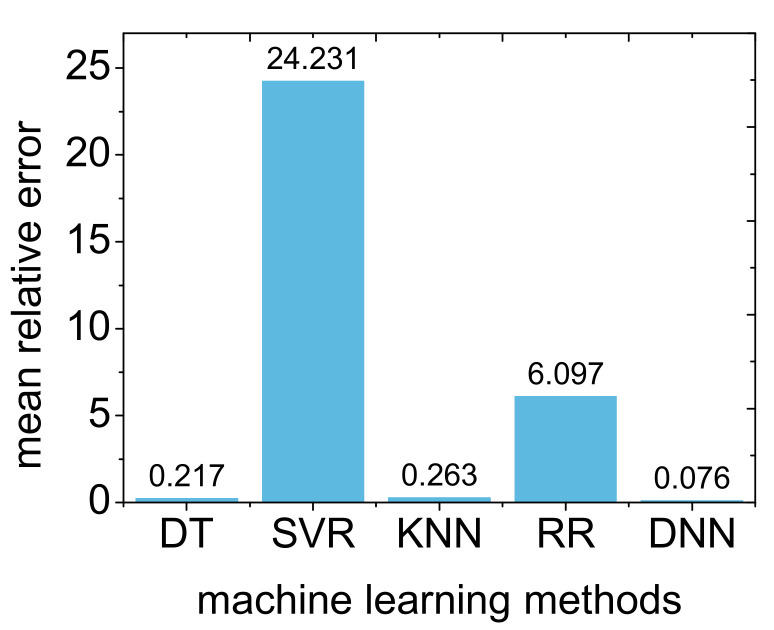
Comparison of the mean relative errors for drain transient current pulses as predicted by the traditional machine learning method and deep neural network method.

We also used traditional machine learning methods to predict the drain transient current pulse. These methods included decision tree (DT), support vector regression (SVR), K-nearest neighbor (KNN), and ridge regression (RR) [35,36,37,38]. We compared the mean relative error predicted by traditional machine learning methods with the prediction result of a deep neural network, as shown in Figure 8. It can be seen that the effect of the decision tree and K-nearest neighbor is good; however, the result is still inferior to that of a deep neural network.

### 3.2. Results on the Prediction of Drain Transient Current Peak and Total Collected Charge

**Figure 9:**
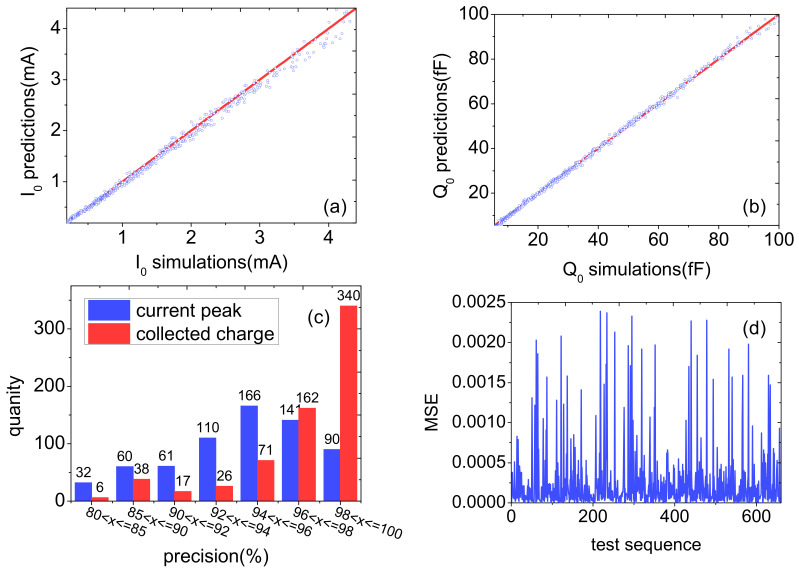
Results for the prediction of drain transient current peak and total collected charge: (**a**) shows the comparison between the simulated value and the predicted value of I_0_; (**b**) shows the comparison between the simulated value and the predicted value of Q_0_; (**c**) is the statistical graph of accuracy distribution of I_0_ and Q_0_; and (**d**) shows the MSE value of the test set.

Figure 9 shows results for the peak values of the drain transient current and total collected charges. Figure 9a shows the comparison between the predicted value and the simulated value of the peak values of the drain transient current in the test set, and Figure 9b shows the comparison between the predicted value and the simulated value of the total collected charge in the test set; the red line is the range of simulation value, and the blue point is the predicted value. It can be seen that the predicted value fluctuates within a small range around the simulation value. Figure 9c is the statistical graph of the accuracy distribution of I_0_ and Q_0_. The accuracy of I_0_ and Q_0_ were both above 80%. The test sets with the accuracy of I_0_ between 94% and 96% accounted for the most data, and the test sets with the accuracy of Q_0_ above 98% accounted for the most data, and the prediction effect was better than I_0_. The average accuracy of I_0_ and Q_0_ in the test set was 94.00% and 96.95%, respectively. Figure 9d shows the mean square error of the test set. It can be seen from the figure that the MSE values are all below 0.0025 and most of them are below 0.0005. The average MSE value for 660 sets of data in the test set was 0.00027.

**Figure 10:**
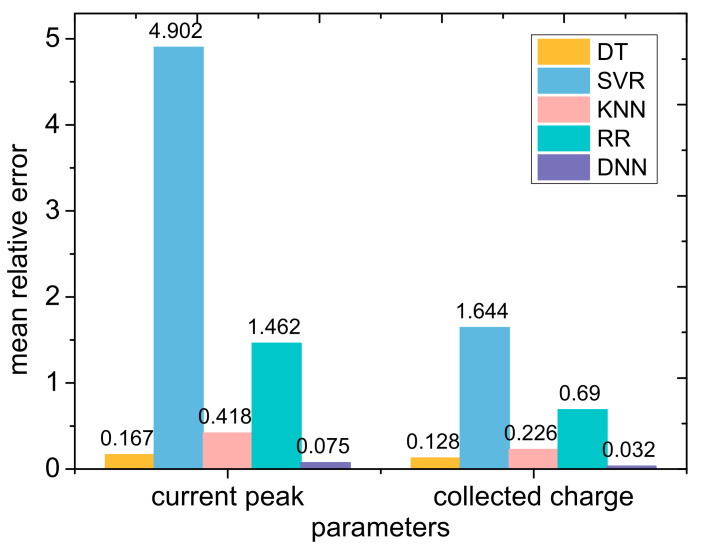
Comparison of the mean relative errors for the peak drain transient current and total collected charge as predicted by traditional machine learning and deep neural network methods.

In the same way, we also used traditional machine learning methods such as DT, SVR, KNN, and RR to predict the peak values of the drain transient current and total collected charges, and compared them with the deep neural network we previously trained; the results are shown in Figure 10. Similar to the results for the transient current pulse, DT and KNN have better prediction performances for current peak and collected charge than other traditional machine learning methods; however, DNN still has the best prediction performance.

## 4. Conclusions

In this paper, we proposed a deep neural network to predict the single-event effect in FDSOI devices. By inputting different LET values, particle incident positions, particle incident directions, and drain bias voltages, the method can quickly and accurately obtain the drain transient current pulse, the peak value of the drain transient current, and the total collected charge. The network model trained by us can predict the peak value of drain transient current and total collected charge with an accuracy of up to 94.00% and 96.95%, respectively, and the goodness of fit for predicting drain transient pulse can reach 0.996. Compared with TCAD Sentaurus, the speed is increased by 5.10 × 10^2^ and 1.38 × 10^3^, respectively. This provides great convenience for the study of the single-event effect of FDSOI devices.

In addition, the device research method based on deep learning provided by us is not only limited to the single-event effect in FDSOI devices but can also be extended to other microelectronic devices, providing a new idea for the research of microelectronic devices.

## Figures and Tables

**Table 1:** FDSOI device parameters.

Parameters	Values
gate length (L)	28 nm
buried oxygen layer thickness (T_box_)	20 nm
gate dielectric layer thickness (T_ox_)	1.2 nm
backplane layer thickness (T_bp_)	25 nm
metal work function (WF)	4.52 eV
backplane doping concentration (N_bp_)	2 × 10^18^ cm^−3^
substrate doping concentration (N_sub_)	1 × 10^14^ cm^−3^
bulk doping concentration (N_bd_)	1 × 10^15^ cm^−3^
source doping concentration (N_s_)	4.4 × 10^20^ cm^−3^
drain doping concentration (N_d_)	4.4 × 10^20^ cm^−3^

**Table 2:** Input parameters of the deep neural network.

Input Parameters	Range/Step
Linear transmission energy (LET)	[10, 100]/10 (MeV·cm^2^/mg)
The incident position of the particle (x)	[−75, 75]/15 (nm)
The incident angle of the particle (θ)	[0, 75]/15 (°)
Drain bias voltage (V_d_)	[0.2, 1]/0.2 (V)

**Table 3:** Partial data of network training and testing.

LET (MeV∙cm^2^/mg)	x (nm)	θ (°)	V_d_ (V)	I_0_ (mA)	Q_0_ (fF)
10	−45	75	0.2	0.168	9.69
10	−45	75	0.4	0.213	11.15
20	60	30	1	1.741	48.93
100	30	15	1	3.697	97.01

## Data Availability

No new data were created or analyzed in this study. Data sharing is not applicable to this article.

## References

[1] Calienes W., Vladimirescu A., Reis R. (2016). Simulation of Single-Event Effects on Fully Depleted Silicon—On—Insulator (FDSOI) CMOS. Semiconductor Devices in Harsh Conditions.

[2] RLiu R., Ferlet-Cavrois V., Evans A., Chen L., Li Y., Glorieux M., Wong R., Wen S.-J., Cunha J., Summerer L. (2017). Single Event Transient and TID Study in 28 nm UTBB FDSOI Technology. IEEE Trans. Nucl. Sci..

[3] Planes N., Weber O., Barral V., Haendler S., Noblet D., Croain D., Haond M. 28 nm FDSOI technology platform for high-speed low-voltage digital applications. Proceedings of the 2012 Symposium on VLSI Technology (VLSIT).

[4] Fenouillet-Beranger C., Denorme S., Perreau P., Buj C., Faynot O., Andrieu F., Skotnicki T. (2008). FDSOI devices with thin BOX and ground plane integration for 32nm node and below. Solid-State Electron..

[5] Peng C., Lei Z., Zhang Z., En Y., Huang Y. Investigating Neutron-Induced Single Event Transient Characteristics by TCAD Simulations in 65 nm Technology and Below. Proceedings of the 2019 3rd International Conference on Radiation Effects of Electronic Devices (ICREED).

[6] Bartra W.C., Vladimirescu A., Reis R. Process and temperature impact on single-event transients in 28nm FDSOI CMOS. Proceedings of the 2017 IEEE 8th Latin American Symposium on Circuits & Systems (LASCAS).

[7] Gouker P.M., Gadlage M.J., McMorrow D., McMarr P., Hughes H., Wyatt P., Keast C., Bhuva B.L., Narasimham B. (2009). Effects of Ionizing Radiation on Digital Single Event Transients in a 180-nm Fully Depleted SOI Process. IEEE Trans. Nucl. Sci..

[8] Wang Q., Liu H., Wang S., Chen S. (2018). TCAD Simulation of Single-Event-Transient Effects in L-Shaped Channel Tunneling Field-Effect Transistors. IEEE Trans. Nucl. Sci..

[9] Chen Y., Hu S.D., Cheng K., Jiang Y., Zhou J., Tang F., Zhou X.C., Gan P. (2016). Improving breakdown performance for novel LDMOS using n + floating islands in substrate. Electron. Lett..

[10] Zeng K., Vaidya A., Singisetti U. (2018). 1.85 kV Breakdown Voltage in Lateral Field-Plated Ga_2_O_3_ MOSFETs. IEEE Electron Device Lett..

[11] Bi J., Li B., Han Z., Luo J., Chen L., Lin-Shi X. 3D TCAD simulation of single-event-effect in n-channel transistor based on deep sub-micron fully-depleted silicon-on-insulator technology. Proceedings of the 2014 12th IEEE International Conference on Solid-State and Integrated Circuit Technology (ICSICT).

[12] Ni T., Guo B., Yang C. Design of Ultrasonic Testing System for Defects of Composite Material Bonding Structure Based on Deep Learning Technology. Proceedings of the 2020 International Conference on Virtual Reality and Intelligent Systems (ICVRIS).

[13] Mun J., Jeong J. Design and Analysis of Optimal Recipe Prediction Model Based on Deep Learning for Advanced Composite Material Injection Molding. Proceedings of the 2021 International Conference on Computer Communication and Artificial Intelligence (CCAI).

[14] Chen Q., Shao T., Xing Y., Zhou Z. Machine Learning-Based Damage Predicion Method for the Micro/Nano Structures Fabricated by Helium Focused Ion Beam. Proceedings of the 2021 21st International Conference on Solid-State Sensors, Actuators and Microsystems (Transducers).

[15] Tang Y., Kojima K., Koike-Akino T., Wang Y., Jha D.K., Parsons K., Qi M. Nano-Optic Broadband Power Splitter Design via Cycle-Consistent Adversarial Deep Learning. Proceedings of the 2021 Conference on Lasers and Electro-Optics (CLEO).

[16] Li C., Yang Y., Liang H., Wu B. (2022). Learning Quantum Drift-Diffusion Phenomenon by Physics-Constraint Machine Learning. IEEE/ACM Trans. Netw..

[17] Chen J., Alawieh M.B., Lin Y., Zhang M., Zhang J., Guo Y., Pan D.Z. (2020). Powernet: SOI Lateral Power Device Breakdown Prediction With Deep Neural Networks. IEEE Access.

[18] Chen J., Guo Y., Lin Y., Alawieh M.B., Zhang M., Zhang J., Pan D.Z. Breakdown Voltage Prediction of SOI Lateral Power Device Using Deep Neural Network. Proceedings of the 2019 Cross Strait Quad-Regional Radio Science and Wireless Technology Conference (CSQRWC).

[19] Mehta K., Wong H.-Y. (2021). Prediction of FinFET Current-Voltage and Capacitance-Voltage Curves Using Machine Learning With Autoencoder. IEEE Electron Device Lett..

[20] Alan M., Aküner M.C., Kepez A. Biosignal Classification and Disease Prediction with Deep Learning. Proceedings of the 2020 Innovations in Intelligent Systems and Applications Conference (ASYU).

[21] Nadell C.C., Huang B., Malof J.M., Padilla W.J. (2019). Deep learning for accelerated all-dielectric metasurface design. Opt. Express.

[22] Sun Y., Liu R., Huang Q., Liu Z., Wang T., Shi Y., Li X. (2021). Analysis of single-event effects in selected BOX-based FDSOI transistor and inverter. Radiat. Phys. Chem..

[23] Xu J., Guo Y., Song R., Liang B., Chi Y. (2019). Supply Voltage and Temperature Dependence of Single-Event Transient in 28-nm FDSOI MOSFETs. Symmetry.

[24] Radu M.D., Costea I.M., Stan V.A. Automatic Traffic Sign Recognition Artificial Inteligence—Deep Learning Algorithm. Proceedings of the 2020 12th International Conference on Electronics, Computers and Artificial Intelligence (ECAI).

[25] Wang L., Xia Y. Artificial Intelligence Brain. Proceedings of the 2021 International Conference on Computer Engineering and Artificial Intelligence (ICCEAI).

[26] Kaplan A., Güldogan E., Çolak C., Arslan A.K. Prediction of Melanoma from Dermoscopic Images Using Deep Learning-Based Artificial Intelligence Techniques. Proceedings of the 2019 International Artificial Intelligence and Data Processing Symposium (IDAP).

[27] Tang H., Liu H., Xiao W., Sebe N. (2021). When Dictionary Learning Meets Deep Learning: Deep Dictionary Learning and Coding Network for Image Recognition With Limited Data. IEEE Trans. Neural Networks Learn. Syst..

[28] Toğaçar M., Ergen B., Özyurt F. Deep learning activities on remote sensed hyperspectral images. Proceedings of the 2018 International Conference on Artificial Intelligence and Data Processing (IDAP).

[29] Burroughs S.J., Gokaraju B., Roy K., Khoa L. DeepFakes Detection in Videos using Feature Engineering Techniques in Deep Learning Convolution Neural Network Frameworks. Proceedings of the 2020 IEEE Applied Imagery Pattern Recognition Workshop (AIPR).

[30] Roy S., Menapace W., Oei S., Luijten B., Fini E., Saltori C., Huijben I., Chennakeshava N., Mento F., Sentelli A. (2020). Deep Learning for Classification and Localization of COVID-19 Markers in Point-of-Care Lung Ultrasound. IEEE Trans. Med. Imaging.

[31] Lai C., Gao Q., Zheng Z., Yuan D., Zhou B., Hong R. Research on Head-up and Down Behavior Computer Detection by Deep Learning and Artificial Intelligence. Proceedings of the 2021 IEEE 3rd International Conference on Civil Aviation Safety and Information Technology (ICCASIT).

[32] Guha R. Improving the Performance of an Artificial Intelligence Recommendation Engine with Deep Learning Neural Nets. Proceedings of the 2021 6th International Conference for Convergence in Technology (I2CT).

[33] Padilla W.J., Nadell C.C., Huang B., Malof J. Accelerated Terahertz Metasurface Design with Deep Learning. Proceedings of the 2020 IEEE International Conference on Plasma Science (ICOPS).

[34] Strickland M., Strickland D., Royston S., Riepnieks A. Frequency Estimation using Curve Fitting. Proceedings of the 2020 9th International Conference on Renewable Energy Research and Application (ICRERA).

[35] Mitrofanov S., Semenkin E. An Approach to Training Decision Trees with the Relearning of Nodes. Proceedings of the 2021 International Conference on Information Technologies (InfoTech).

[36] Zou J., Li C., Yang Q., Li Q. Fault prediction method based on SVR of improved PSO. Proceedings of the 27th Chinese Control and Decision Conference (2015 CCDC).

[37] Bajpai D., He L. Evaluating KNN Performance on WESAD Dataset. Proceedings of the 2020 12th International Conference on Computational Intelligence and Communication Networks (CICN).

[38] Maalouf M., Khoury N., Homouz D., Polychronopoulou K. Accurate Prediction of Gas Compressibility Factor using Kernel Ridge Regression. Proceedings of the 2019 Fourth International Conference on Advances in Computational Tools for Engineering Applications (ACTEA).

